# Waterborne diseases.

**DOI:** 10.3201/eid0707.017723

**Published:** 2001

**Authors:** P R Hunter, J M Colford, M W LeChevallier, S Binder, P S Berger

**Affiliations:** World Health Organization, Geneva, Switzerland.


### Waterborne Diseases

**Paul R. Hunter,* Jack M. Colford,† Mark W. LeChevallier‡**
**Sue Binder,‡ and Paul S. Berger§**
*World Health Organization, Geneva, Switzerland; †National Institutes of Health, Bethesda, Maryland, USA; ‡Centers for Disease Control and Prevention, Atlanta, Georgia, USA; and §U.S. Environmental Protection Agency, Washington, DC, USA

### Waterborne Disease Outbreaks

In the United States, 127 drinking water outbreaks, most of them associated with groundwater systems, were reported to CDC from 1990 through 1998. The number of outbreaks has declined over the last 20 years, probably as a result of actions by the U.S. Environmental Protection Agency (EPA), water utilities, and public health officials; however, changes in reporting practices may also have contributed to this trend.

### World Water Issues

Paul R. Hunter, consultant medical microbiologist and director of the Chester Public Health Laboratory and honorary professor of epidemiology and public health at the University of Central Lancashire, presented World Health Organization data that showed high morbidity and death rates worldwide due to consumption of unsafe drinking water. Currently, about 20% of the world's population lacks access to safe drinking water, and more than 5 million people die annually from illnesses associated with unsafe drinking water or inadequate sanitation. If everyone had safe drinking water and adequate sanitation services, there would be 200 million fewer cases of diarrhea and 2.1 million fewer deaths caused by diarrheal illness each year.

Dr. Hunter noted the wide variety of microbes recognized since 1980 as waterborne disease agents, including *Cryptosporidium*, *Cyclospora*, *Escherichia coli* O157:H7, *Legionella*, *Helicobacter pylori*, hepatitis E virus, *Toxoplasma*, and others. The factors that contribute to the emergence and spread of disease agents are ecologic changes (including those caused by human activity), international travel and commerce, technology, human demographics and behavior, microbial evolution, and the breakdown of public health systems. Dr. Hunter warned that global freshwater consumption rose sixfold between 1900 and 1995, and that this places increasing stress on available drinking water reserves. This increasing stress will result in ecologic damage from over-extraction from rivers, saltwater intrusion into groundwater from over-extraction of groundwater, more highly contaminated water sources, and the potential struggle for access to water. Dr. Hunter concluded his presentation by discussing the threat of biological terrorism via microbes that could be used for deliberate contamination of the water supply.

Address for correspondence: Sue Binder, Centers for Disease Control and Prevention, 4770 Buford Highway, NE, Mailstop K02, Atlanta, GA 30341, USA; fax: 770-488-4422; e-mail: sbinder@cdc.gov

### Methodologic Issues in the Evaluation of Waterborne Disease

Jack M. Colford, assistant professor of epidemiology, at the University of California-Berkeley, discussed methods for estimating the incidence of infectious diseases attributable to the consumption of tap water. In a previously published study, investigators in Canada compared the incidence of gastroenteritis in homes with and without a reverse-osmosis filter. The study showed that 35% of gastrointestinal illness in the community studied was attributable to drinking water. The study was randomized, but participants knew in which group they were enrolled. As a partial consequence of this study, when Congress amended the Safe Drinking Water Act in 1996, it required that EPA and CDC develop a national estimate of waterborne disease occurrence in the United States. In response, CDC and EPA jointly convened a series of workshops and consensus panel meetings to develop an approach to meet this mandate.

As a result of these meetings, EPA and CDC are supporting several studies, the largest of which will be a randomized, blinded, placebo-controlled trial, involving treatment of in-house drinking water. A pilot study of this intervention trial was recently conducted with residents in 74 homes in northern California; some residents received an active water treatment device containing a 1-µm filter and ultraviolet (UV) light for disinfection, and others received a placebo—the same device without a filter or UV light. Results from this study and a similar study in Australia should be released in fall 2000. Other studies employing the same design will include persons using a groundwater system in Davenport, Iowa; HIV-positive persons in San Francisco; and elderly persons in Sonoma, California. The results of these studies will be used to estimate the percentage of gastrointestinal disease associated with different types of drinking water. When compared with the total prevalence of gastrointestinal disease in the United States, these results will provide an estimate of the national burden of waterborne disease caused by drinking water.

### Biofilms

Mark W. LeChevallier, director of research at the American Water Works Service Company, discussed health concerns regarding biofilms in the drinking water distribution system. Biofilms are coatings of organic and inorganic materials in pipes that can harbor, protect, and allow the proliferation of several bacterial pathogens, including *Legionella* and *Mycobacterium avium* complex (MAC). Factors that affect bacterial growth on biofilms include water temperature, type of disinfectant and residual concentration, assimilable organic carbon level, biodegradable organic carbon level, degree of pipe corrosion, and treatment/distribution system characteristics. Chloramine is considerably more effective than chlorine for controlling *Legionella* in biofilms, presumably because chloramine is more stable and thus less reactive than chlorine, allowing it to penetrate the biofilm more deeply.

An important factor in distribution system contamination and bacterial growth on biofilms is transient water pressure fluctuations that create pressure waves that pass through pipes in the distibution system. During the negative portion of the pressure wave, a substantial amount of contaminated water (>1 gal per minute) from the outside can be pulled into pipes through a small leak. This problem is aggravated when sewer lines are placed close to water pipes. Dr. LeChevallier stated that a number of waterborne disease outbreaks have been linked to distribution system deficiencies. Among the agents of nosocomial waterborne disease is MAC. This opportunistic bacterial pathogen lives in water, is resistant to water disinfection (much more so than *Giardia* cysts), and grows in pipe biofilms.

### Xenotransplantation: Benefits and Risks

**Louisa Chapman**
Centers for Disease Control and Prevention, Atlanta, Georgia, USA

During transitional periods new scientific understandings bring new questions. The pivotal issues in xenotransplantation concern biohazards. The U.S. Public Health Service defines xenotransplantation as ". . . any procedure that involves the transplantation, implantation, or infusion into a human recipient of either A) live cells, tissues, or organs from a nonhuman animal source or B) human body fluids, cells, tissues, or organs that have had ex vivo contact with live nonhuman animal cells, tissues, or organs."

Prior to 1990, xenotransplants were largely whole organs; recipients survived only days or weeks. However, in most recent xenotransplantation trials, immunoprotected porcine neurologic, pancreatic, and hepatic cells are used to treat degenerative neurologic disorders, diabetes, or hepatic failure. Increasingly, xenotransplantation products function for prolonged periods in recipients who survive months or years.

Xenotransplantation is a public health concern because it has the potential to infect human recipients with agents that do not ordinarily infect humans, thereby introducing new infections to humans. Therefore, xenotransplantation combines a potential benefit with a potential risk to humans that is presently unknown.

Xenogeneic infections belong to a larger category of "bioproduct-acquired" infections, an example of which is simian virus 40 (SV40). SV40, a polyomavirus, contaminated polio vaccine stocks in the 1950s. Investigations into whether SV40 infection is associated with an increased risk of cancer have been inconclusive. This lingering uncertainty about the long-term significance of apparently innocuous persistent human infection with nonhuman viruses underscores the potential for therapeutic use of bioproducts to have unintended consequences.

The PHS Guideline on Infectious Disease Issues in Xenotransplantation describes a system of safeguards built around two key concepts: pretransplant screening of some animal herds, source animals, and xenotransplantation products to minimize the risk of xenogeneic infections with recognized pathogens and posttransplant surveillance of recipients for previously unrecognized xenogeneic organisms.

Endogenous retroviruses exist as inactive proviral DNA in the germline of all mammals adequately studied to date. However, inactive genomic endogenous retroviruses can often express active virus capable of infecting human cell lines in vitro. Thus, xenotransplantation products contain benign genomic DNA that, on transfer into a human, may express infectious retrovirus capable of creating active, persistent infection. The importance of this infectious potential of animal tissue devoid of any identifiable exogenous microorganisms has been the subject of much concern and scientific inquiry over the past 5 years.

To date, limited studies on humans exposed to pig cells and tissues have produced no evidence of porcine endogenous retrovirus infection. However, the persistent presence of microchimeric xenogeneic pig cells in the human recipient confirms that even temporary exposure to xenotransplantation products may continuously expose humans to infectious agents contained within them. For this reason, it is argued that humans should not be exposed to xenotransplantation products containing infectious agents whose ability to infect, cause disease in, or be spread among humans is incompletely defined.

Xenotransplantation is a process that occurs under controlled circumstances; thus, measures can be implemented to minimize associated iatrogenic biohazards. Studies performed in the service of developing policies on xenotransplantation can model other approaches to science-based risk minimization used for other bioproducts.

Address for correspondence: Louisa Chapman, Centers for Disease Control and Prevention, 1600 Clifton Road, Mailstop G19, Atlanta, GA 30333, USA; fax: 404-639-0868; e-mail: lec3@cdc.gov

